# Genomic Features, Comparative Genomics, and Antimicrobial Susceptibility Patterns of *Elizabethkingia bruuniana*

**DOI:** 10.1038/s41598-019-38998-6

**Published:** 2019-02-19

**Authors:** Jiun-Nong Lin, Chung-Hsu Lai, Chih-Hui Yang, Yi-Han Huang, Hsi-Hsun Lin

**Affiliations:** 1 School of Medicine, College of Medicine, I-Shou University, Kaohsiung, Taiwan; 2 Division of Infectious Diseases, Department of Internal Medicine, E-Da Hospital, I-Shou University, Kaohsiung, Taiwan; 3 Department of Critical Care Medicine, E-Da Hospital, I-Shou University, Kaohsiung, Taiwan; 4 Department of Biological Science and Technology, Meiho University, Pingtung, Taiwan; 5 General Clinical Research Center, Department of Medical Research, Taipei Veterans General Hospital, Taipei, Taiwan; 6 School of Medicine, National Yang-Ming University, Taipei, Taiwan

## Abstract

*Elizabethkingia bruuniana* is a novel species of the *Elizabethkingia* genus. There is scant information on this microorganism. Here, we report the whole-genome features and antimicrobial susceptibility patterns of *E*. *bruuniana* strain EM798-26. *Elizabethkingia* strain EM798-26 was initially identified as *E*. *miricola*. This isolate contained a circular genome of 4,393,011 bp. The whole-genome sequence-based phylogeny revealed that *Elizabethkingia* strain EM798-26 was in the same group of the type strain *E*. *bruuniana* G0146^T^. Both *in silico* DNA-DNA hybridization and average nucleotide identity analysis clearly demonstrated that *Elizabethkingia* strain EM798-26 was a species of *E*. *bruuniana*. The pan-genome analysis identified 2,875 gene families in the core genome and 5,199 gene families in the pan genome of eight publicly available *E*. *bruuniana* genome sequences. The unique genes accounted for 0.2–12.1% of the pan genome in each *E*. *bruuniana*. A total of 59 potential virulence factor homologs were predicted in the whole-genome of *E*. *bruuniana* strain EM798–26. This isolate was nonsusceptible to multiple antibiotics, but susceptible to aminoglycosides, minocycline, and levofloxacin. The whole-genome sequence analysis of *E*. *bruuniana* EM798-26 revealed 29 homologs of antibiotic resistance-related genes. This study presents the genomic features of *E*. *bruuniana*. Knowledge of the genomic characteristics provides valuable insights into a novel species.

## Introduction

*Elizabethkingia* is a genus of aerobic, gram-negative, nonmotile, non-spore-forming, and non-fermenting bacilli^1^. These microorganisms are extensively distributed in soil, water, and plants, but they do not normally exist in human microflora^1–3^. Among the members of this genus, the type species, *E*. *meningoseptica*, is the most well-known species that causes human infections since its first identification by Elizabeth O. King in 1959^4^. The second species of the genus, *E*. *miricola*, was isolated in 2003 from condensation water on the space station Mir^5^. The third species, *E*. *anophelis*, was first recognized from the midgut of the mosquito *Anopheles gambiae* in 2011^6^. Three new species, namely, *E*. *bruuniana*, *E*. *ursingii*, and *E*. *occulta*, were proposed to be novel members of the *Elizabethkingia* genus in 2017^1^. As of now, six species are included in the *Elizabethkingia* genus. A noteworthy rise in the lethal infections associated with this genus has been identified worldwide recently^7–12^.

We previously published the complete genome sequence of the *E*. *miricola* strain EM798-26 isolated from the blood of a cancer patient (GenBank accession number CP023746)^13^. This isolate was initially identified as *E*. *miricola* using 16S ribosomal RNA (rRNA) gene sequencing, which showed a 99.9% identity to *E*. *miricola* ATCC 33958 and 99.6% identity to *E*. *miricola* BM10. After the proposal of the three novel *Elizabethkingia* species, we revisited the taxonomy of the *E*. *miricola* strain EM798-26 using *in silico* DNA-DNA hybridization (DDH) and average nucleotide identity (ANI) analysis based on whole-genome sequences. In this study, we reported the emendation of the strain EM798-26 as a later subjective synonym of *E*. *bruuniana*. We then investigated the genomic features and phylogenetic diversity of *E*. *bruuniana* isolates available in the National Center for Biotechnology Information (NCBI) genome sequence repository. We finally described the antimicrobial susceptibility patterns of *E*. *bruuniana* strain EM798-26.

## Materials and Methods

### Ethics and experimental biosafety statements

This study was approved by the Institutional Review Board of E-Da Hospital (EMRP-106-105). The need for patient’s informed consent was waived by the Institutional Review Board of E-Da Hospital as the retrospective analysis of anonymously clinical data posed no more than minimal risk of harm to subjects and involved no procedures for which written consent was normally required outside of the research context. The experiments in this study were approved by the Institutional Biosafety Committee of E-Da Hospital. All experiments were performed in accordance with relevant guidelines and regulations.

### Isolate of Elizabethkingia strain EM798-26

*Elizabethkingia* strain EM798-26 was isolated from the blood of an 81-year-old male patient with diffuse large B-cell lymphoma. This patient was admitted due to neutropenic fever after chemotherapy for lymphoma. The blood culture was performed using BacT/ALERT 3D Microbial Identification System (bioMérieux, Marcy l’Etoile, France). This isolate was initially identified as *E*. *meningoseptica* using VITEK matrix-assisted laser desorption ionization–time of flight mass spectrometry (bioMérieux) by the clinical microbiology laboratory, and then it was stored as glycerol stocks at −80 °C until use. For experiments, the thawed isolate of strain EM798-26 was subcultured on tryptic soy agar with 5% sheep blood (Becton, Dickinson and Company, Franklin Lakes, NJ, USA). The total DNA of fresh colonies was extracted using a Wizard Genomic DNA Purification Kit (Promega, Madison, WI, USA).

### 16S rRNA sequencing and phylogenetic analysis

The primers and protocols for amplification and sequencing of 16S rRNA gene are listed in Table1^14,15^. To evaluate the phylogenetic diversity between *Elizabethkingia* and other genera, the 16S rRNA gene sequences of the type species and common species of different gram-negative genera were compared (Supplementary Table S1). The sequences were aligned using the ClustalW function with default options in the MEGA software^16^. Genetic relationships were calculated using the neighbor-joining method based on Kimura 2-parameter distances in the MEGA software^16^. Phylogenetic trees were re-constructed in the Dendroscope software^17^.

**Table 1:** Primers of PCR and sequencing for 16S rRNA in this study.

Primer	Sequence (5′ to 3′)	Amplicon size (bp)	References
**PCR of 16S rRNA**			
8 f	CACGGATCCAGACTTTGAT(C/T)(A/C)TGGCTCAG	1498	14
1512r	GTGAAGCTTACGG(C/T)TAGCTTGTTACGACTT		
**Sequencing of 16S rRNA**			
8 f	CACGGATCCAGACTTTGAT(C/T)(A/C)TGGCTCAG	—	15
534r	ATTACCGCGGCTGCTGG		
534 f	CCAGCAGCCGCGGTAAT		
968 f	AACGCGAAGAACCTTAC		
1512r	GTGAAGCTTACGG(C/T)TAGCTTGTTACGACTT		

### Whole-genome sequencing

The whole genome of the strain EM798-26 was sequenced using Illumina HiSeq2000 (Illumina, San Diego, CA, USA) and PacBio (Pacific Biosciences, Menlo Park, CA, USA) sequencing platforms as our previous report^13^. The genome was then hybrid assembled, and structural errors were corrected by optical mapping (Bionano Genomics, San Diego, CA, USA).

### Whole-genome phylogenetic analysis and species identification

To determine the phylogenetic origin with respect to other *Elizabethkingia* strains, the whole-genome sequences of 14 publicly published “*E*. *miricola*” strains and each type strain of *E*. *meningoseptica* KC1913^T^ (=ATCC 13253 ^T^), *E*. *miricola* GTC 862 ^T^ (=KCTC 12492 ^T^ = W3-B1), *E*. *anophelis* R26^T^, *E*. *bruuniana* G0146^T^, *E*. *ursingii* G4122^T^, and *E*. *occulta* G4070^T^ were compared (Supplementary Table S2). The whole-genome sequence-based phylogenetic tree was constructed using the online pipeline Reference Sequence Alignment Based Phylogeny Builder (REALPHY)^18^. To confirm the species of *Elizabethkingia*, we calculated the *in silico* DDH and ANI values using Genome-to-Genome Distance Calculator (GGDC)^19^ and OrthoANI^20^, respectively. An ANI cutoff value of 95% and a DDH cutoff value of 70% was used as species delimitation criteria^19,21^. The heat maps were generated using CIMminer (https://discover.nci.nih.gov/cimminer/).

### Genome annotation and analysis

The assembled genome was submitted to the NCBI Prokaryotic Genome Annotation Pipeline^22^ and the Rapid Annotations based on Subsystem Technology (RAST) Prokaryotic Genome Annotation Server (http://rast.nmpdr.org/)^23,24^ for genome annotations. The pan genome and core genome were analyzed using the Bacterial Pan Genome Analysis (BPGA) pipeline^25^. The graphical map of the circular genome was generated using the CGView Server (http://stothard.afns.ualberta.ca/cgview_server/)^26^. The virulence factors of the strain EM798-26 were predicted using the Virulence Factor Database (VFDB, http://www.mgc.ac.cn/VFs/)^27^. Antibiotic resistance genes were searched using the Antibiotic Resistance Genes Database BLAST Server (ARDB, https://ardb.cbcb.umd.edu/)^28^. An expectation value <1e-5 and ≥30% identity of the homologs were used as a threshold of the BLASTP searches^29^.

### Antimicrobial susceptibility testing

The minimum inhibitory concentration (MIC) was examined using the broth microdilution method (Thermo Fisher Scientific/Trek Diagnostics Systems, Oakwood Village, OH, USA). The susceptibilities were determined according to the interpretive standards for “other non*-Enterobacteriaceae*” as suggested by the Clinical and Laboratory Standards Institute (CLSI) guideline^30^. The MIC of tigecycline was interpreted according to the *Enterobacteriaceae* susceptibility breakpoints of the USA FDA (susceptible MIC, ≤2 mg/L; intermediate MIC, 4 mg/L; resistant MIC, ≥8 mg/L)^31^.

## Results and Discussion

### Phylogenetic relationships between Elizabethkingia and other genera

**Figure 1:**
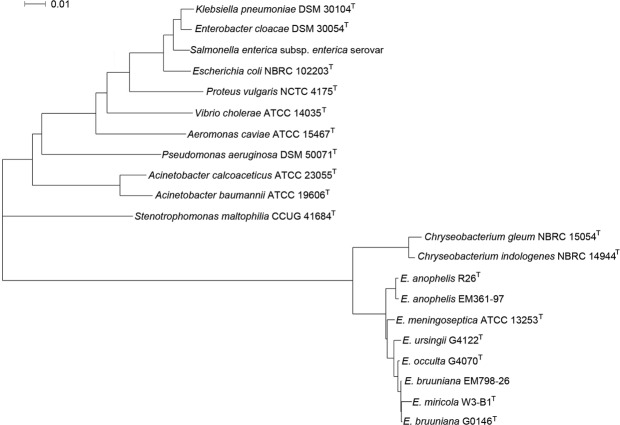
Phylogenetic analysis of 16S rRNA gene sequencing using the neighbor-joining method based on Kimura 2-parameter distances. The tree was constructed from the 16S rRNA gene sequences of *E*. *bruuniana* EM798-26 and the respective reference sequences from GenBank. The scale length indicates 0.01 nucleotide substitutions per nucleotide site.

The phylogenetic analysis of 16S rRNA sequences between the *Elizabethkingia* species and the respective reference sequences of other gram-negative genera is shown in Fig. 1. On the basis of 16S rRNA gene sequencing, *E*. *meningoseptica* ATCC 13253 ^T^, *E*. *anophelis* R26^T^, *E*. *anophelis* EM361-97 (our previously published genome^9^), *E*. *miricola* W3-B1^T^, *E*. *bruuniana* G0146^T^, *E*. *bruuniana* EM798-26, *E*. *ursingii* G4122^T^, and *E*. *occulta* G4070^T^ were in the same branch of phylogenetic tree.

*Chryseobacterium* species were closest to *Elizabethkingia* species, and shared a recent common ancestor with *Elizabethkingia*. Both genera belong to the *Flavobacteriaceae* family. The phylogenetic branch of *Stenotrophomonas maltophilia*, *Acinetobacter baumannii*, and *Pseudomonas aeruginosa* were near to the branch of *Elizabethkingia* and *Chryseobacterium*. All these genera are glucose non-fermenting gram-negative bacilli. In contrast, the microorganisms of *Enterobacteriaceae* family, including *Klebsiella pneumoniae*, *Enterobacter cloacae*, *Salmonella enterica*, *Escherichia coli*, and *Proteus vulgaris* were farthest away from *Elizabethkingia* in the phylogenetic tree based on 16S rRNA gene sequences.

### Genome description of strain EM798-26

**Figure 2:**
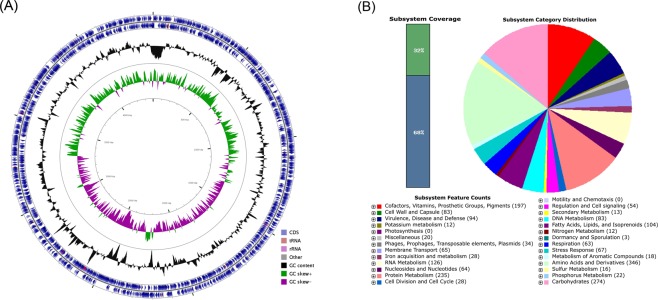
Genomic features of *Elizabethkingia bruuniana* EM798-26. (**A**) The genome of strain EM798-26 contained 3,877 protein-coding genes and 80 pseudogenes. There were 72 RNA genes, including 15 ribosomal RNAs (rRNAs), 54 transfer RNAs (tRNAs), and three noncoding RNAs (ncRNAs). The outer two circles demonstrate the coding sequence (CDS), tRNA, and rRNA. The third circle shows the GC content (black). The fourth circle represents the GC skew curve (positive GC skew, green; negative GC skew, violet). (**B**) The genome of *E*. *bruuniana* EM798-26 annotated using the Rapid Annotation System Technology (RAST) Server. The genome could be classified into 27 categories and 360 subsystems. The green part in the bar chart at the leftmost position corresponds to the percentage of proteins included. The pie chart and count of the subsystem features in the right panel demonstrate the percentage distribution and category of the subsystems.

The total length of the assembled genome was 4,393,011 bp, with a mean G + C content of 35.73%. The genome coverage rate was 220.0× . The statistics of the assembly and annotation are shown in Table 2. The genome contained 3,877 protein-coding genes and 80 pseudogenes. The number of RNA genes was 72, including 15 rRNAs, 54 transfer RNAs (tRNAs), and three noncoding RNAs (ncRNAs) (Fig. 2A). These 3,877 genes could be classified into 27 categories and 360 subsystems (Fig. 2B). Of these subsystems, “amino acids and derivatives” was the largest and accounted for 346 genes, followed by “carbohydrates” (274 genes), “protein metabolism” (235 genes), and “cofactors, vitamins, prosthetic groups, pigments” (197 genes). In the category of “virulence, disease and defense”, 85 genes were related to “resistance to antibiotics and toxic compounds”, including “resistance to vancomycin” (1 gene), “multidrug resistance, tripartite systems found in gram-negative bacteria” (9 genes), “resistance to fluoroquinolones” (4 genes), “β-lactamase” (17 genes), and “multidrug resistance efflux pumps” (16 genes). The high number of antimicrobial resistance homologs suggests that *Elizabethkingia* strain EM798-26 might be a multidrug-resistant strain.

**Table 2:** Assembly and annotation statistics.

Content	Number
Genome size (bp)	4,393,011
Gene number	4,117
Gene length (bp)	3,902,358
GC content (%)	35.73
GC content in gene region (%)	36.68
Gene length/genome (%)	88.83
Gene average length (bp)	948
Intergenic region length (bp)	490,655
GC content in intergenic region (%)	28.17
Intergenic region length/genome (%)	11.17
N50 (bp)	483,147
L50 (bp)	4
Tandem repeat number	118
Tandem repeat length (bp)	11,030
Tandem repeat size (bp)	3–736
Tandem repeat length/genome (%)	0.2511
Genes (total)	4,029
CDS (total)	3,957
Genes (coding)	3,877
Genes (RNA)	72
ribosomal RNA (rRNA)	15
transfer RNA (tRNA)	54
noncoding RNA (ncRNA)	3
Pseudogenes	80

### Whole-genome sequence-based identification of Elizabethkingia species

**Figure 3:**
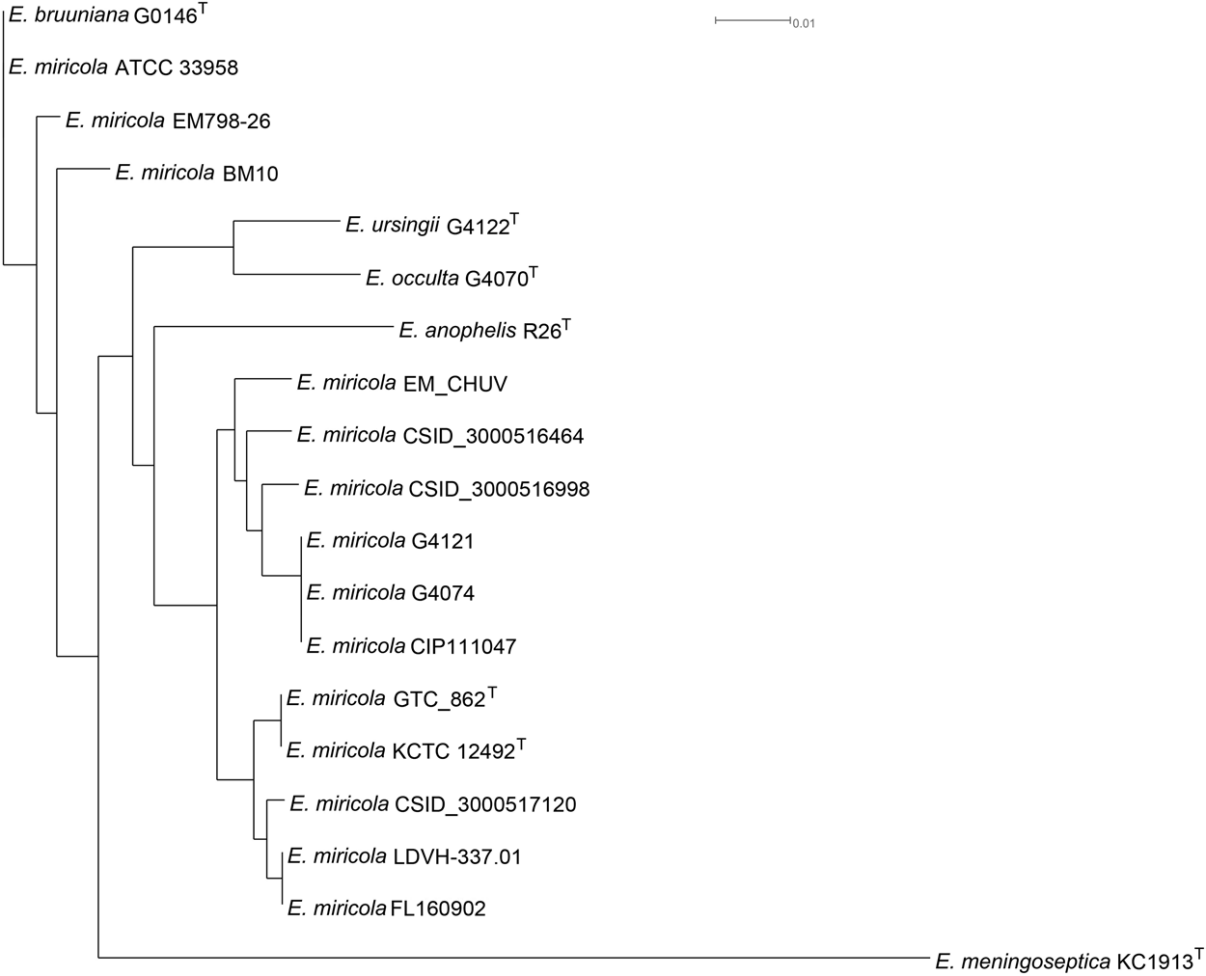
The whole-genome sequence-based phylogenetic tree constructed using the Reference Sequence Alignment Based Phylogeny Builder (REALPHY).

**Figure 4:**
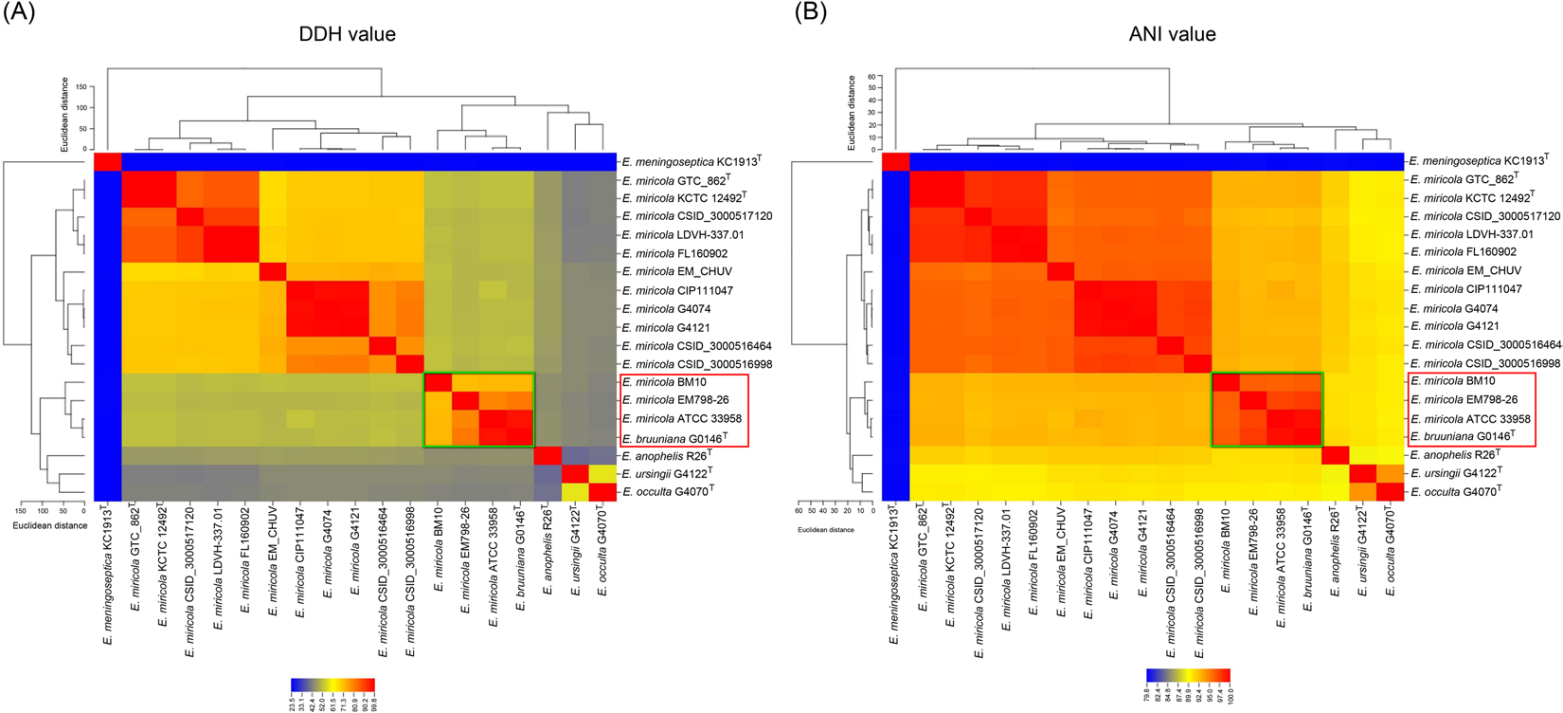
Species identification based on whole-genome sequencing. (**A**) *in silico* DNA-DNA hybridization (DDH) using the Genome-to-Genome Distance Calculator (GGDC). (**B**) Average nucleotide identity (ANI) analysis using OrthoANI.

The whole-genome sequence-based phylogenetic tree was constructed and demonstrated that strains EM798-26, ATCC 33958, and BM10 were in the same genomic group with the type strain *E*. *bruuniana* G0146^T^ (Fig. 3). Both *in silico* DDH (Fig. 4A) and ANI analysis (Fig. 4B) clearly revealed that strains EM798-26, ATCC 33958, BM10, and *E*. *bruuniana* G0146^T^ belonged to the same species.

Before the proposal of the three new species in the *Elizabethkingia* genus, substantial sequence variability in the whole-genome sequences of the *E*. *miricola* strains has been identified and taxonomic re-classification of some strains has been suggested^32,33^. Based on the whole-genome sequence study, Nicholson *et al*. proposed that strains in genomospecies 3 as *E*. *bruuniana* sp. nov. and those in genomospecies 4 as *E*. *ursingii* sp. nov. and *E*. *occulta* sp. nov.^1^. At the time of proposing the novel species of *Elizabethkingia*, Nicholson *et al*. also re-classified *E*. *miricola* ATCC 33958 and *E*. *miricola* BM10 into the species of *E*. *bruuniana* based on the results of the whole-genome DDH, optical maps, and matrix-assisted laser desorption ionization-time of flight mass spectrometry^1^. Similar to the strains ATCC 33958 and BM10, our study clearly demonstrated that strain EM798-26 should also be re-assigned to the species of *E*. *bruuniana*, but not *E*. *miricola*.

### Pan-genome comparisons

**Figure 5:**
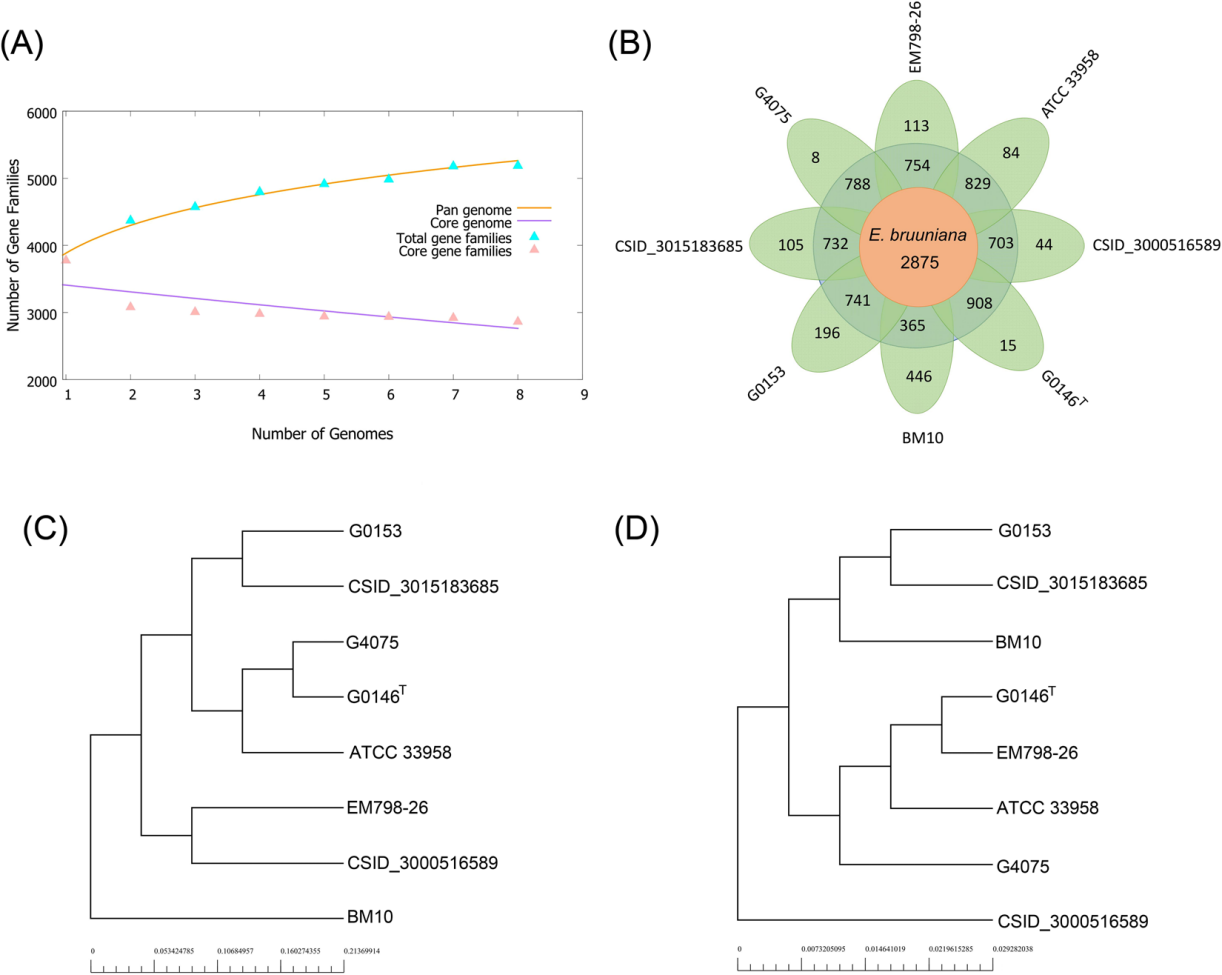
Pan-genome analysis of eight *E*. *bruuniana* isolates in the repertoire of GenBank. (**A**) Pan-genome and core genome plot shows the progression of the pan (orange line) and core (purple line) genomes as more genomes are added for analysis. The pan genome is still open, as the new additional genome significantly increases the total repertoire of genes. Extrapolation of the curve indicates that the gene families in pan genome increased from 3,884 to 5,199, and those in core genome decreased from 3,511 to 2,875. (**B**) Flower plot shows the numbers of core genes (inner circle), accessory genes (middle circle), and unique genes (outer circle). (**C**) Phylogenetic tree based on the pan genome. (**D**) Phylogenetic tree based on the core genome.

Pan-genome analysis has been applied in the evaluation of the genome diversity, genome dynamics, species evolution, pathogenesis, and other features of microorganisms^34^. To better understand the phylogenetic relationship and bacterial evolution, we performed pan-genome analysis of eight publicly available whole-genome sequences of *E*. *bruuniana* isolates (Fig. 5; Supplementary Table S2). The evolution of the pan and core genome is presented in Fig. 5A. As the addition of each new genome sequence of *E*. *bruuniana*, the number of gene families in the pan genome increased from 3,884 to 5,199, and that of gene families in the core genome decreased from 3,511 to 2,875 (Fig. 5A,B). The core genome accounted for on average 55.3% of the pan genome. The gene families of the pan genome represent the housing capacity of the genetic determinants and those of the core genome are usually related to bacterial replication, translation, and maintenance of cellular homeostasis^34,35^. In our study, the unique genes of each *E*. *bruuniana* strain exhibited a wide distribution, ranging from 8 (0.2%) to 446 (12.1%). These unique genes are under relaxed mutation pressure^34,36^ and usually have an association with the pathogenicity and virulence of the microorganisms^34^. Phylogeny based on the pan genome demonstrated that *E*. *bruuniana* EM798-26 was closer to *E*. *bruuniana* CSID_3000516589 (Fig. 5C). In contrast, the tree based on the core genome showed that strain EM798-26 was at a position near the type strain *E*. *bruuniana* G0146^T^ (Fig. 5D). These findings suggest the diverse genetic evolution of the pan and core genomes in different *E*. *bruuniana* strains.

### Potential virulence factors

A total of 59 potential virulence factor homologs were predicted by VFDB with the criteria of ≥30% identity and <1e-5 expectation value (Supplementary Table S3). These genes conferred biofilm formation, capsule polysaccharide synthesis, inhibition of the alternative complement pathway and complement-mediated opsonophagocytosis, iron siderophore synthesis, superoxide dismutase expression, prevention of phagocytosis, prevention of antibody-mediated opsonization, and other functions. These virulence factor homologs in the *E*. *bruuniana* strain EM798-26 are also commonly found in other *Elizabethkingia* species, such as *E*. *anophelis*^9,29^. However, these potential virulence factors need more experiments to prove their pathogenicity.

### Antimicrobial susceptibility testing and antimicrobial resistance-associated genes

The antimicrobial susceptibility testing of *E*. *bruuniana* strain EM798-26 is shown in Table 3. This isolate was non-susceptible to all tested β-lactams, β-lactam-lactamase inhibitors, and carbapenems, but susceptible to gentamicin, amikacin, minocycline, and levofloxacin.

**Table 3:** Antimicrobial susceptibility of *E*. *bruuniana* strain EM798-26 and *E*. *anophelis* strain EM361-97.

Antimicrobial agent	*E*. *bruuniana* strain EM798-26		*E*. *anophelis* strain EM361-97	
	MIC (mg/L)	Interpretation	MIC (mg/L)	Interpretation
Piperacillin	>64	R	32	I
Piperacillin-tazobactam	32/4	I	16/4	S
Ticarcillin-clavulanic acid	>64/2	R	>64/2	R
Ceftazidime	>16	R	>16	R
Cefepime	>32	R	32	R
Ceftriaxone	>32	R	>32	R
Aztreonam	>16	R	>16	R
Imipenem	>8	R	>8	R
Meropenem	>8	R	>8	R
Gentamicin	4	S	>8	R
Tobramycin	>8	R	>8	R
Amikacin	16	S	>32	R
Tetracycline	>8	R	>8	R
Minocycline	<1	S	<1	S
Tigecycline	2	S	2	S
Ciprofloxacin	2	I	>2	R
Levofloxacin	<1	S	>8	R
Trimethoprim-sulfamethoxazole	>4/76	R	>4/76	R

Abbreviations: MIC, minimum inhibitory concentration; *S*, *s*usceptible; I, intermediate; R, resistant.

We compared the antimicrobial susceptibility patterns between *E*. *bruuniana* strain EM798-26 and *E*. *anophelis* strain EM361-97 which was published in our previous study^9^. Both strains demonstrated resistance to multiple antibiotics, but they exhibited susceptibility to minocycline (MIC < 1 mg/L) and tigecycline (MIC = 2 mg/L). However, *E*. *bruuniana* strain EM798-26 was susceptible to levofloxacin, but *E*. *anophelis* strain EM361-97 was resistant to this antimicrobial agent.

Previous studies showed that *Elizabethkingia* isolates were usually resistant to many antimicrobial agents. For example, *E*. *anophelis* and *E*. *meningoseptica* isolated from Hong Kong^8^, the USA^11^, and South Korea^37^ demonstrated high resistance to most β-lactams, including ceftazidime, ceftriaxone, and imipenem, but variable susceptibility to piperacillin-tazobactam, cefepime, ciprofloxacin, and levofloxacin. However, there have been no previous studies describing the antimicrobial susceptibility of *E*. *bruuniana*. Our study first demonstrated the antimicrobial susceptibility testing patterns of *E*. *bruuniana*. As there was only one isolate in our study, further large-scale studies are necessary to investigate the antimicrobial susceptibility pattern of *E*. *bruuniana*.

We investigated the antimicrobial resistance-associated genes using the Antibiotic Resistance Genes Database BLAST Server with a threshold of ≥30% identity and <1e-5 expectation value. The whole-genome sequence analysis of *E*. *bruuniana* EM798-26 revealed 29 homologs of antibiotic resistance-related genes (Supplementary Table S4). These antibiotic resistance genes included β-lactamases, multidrug resistance efflux pumps (aminoglycoside and macrolide), NADP-requiring oxidoreductase, dihydrofolate reductase, undecaprenyl pyrophosphate phosphatase, sulfonamide-resistant dihydropteroate synthase, VanA, B, E, and G vancomycin resistance operon genes, the adenosine triphosphate-binding cassette (ABC) superfamily, the resistance-nodulation-division (RND) family, the major facilitator superfamily (MFS), and the small multidrug resistance (SMR) family. The whole-genome analysis suggested that *E*. *bruuniana* EM798-26 could be a strain resistant to multiple antibiotics. The manifestation of multidrug resistance is compatible with the antimicrobial susceptibility testing of this isolate.

## Conclusions

This study presents the species identification, genomic features, and antimicrobial susceptibility patterns of *E*. *bruuniana*. There is no similar study to describe the genomic features and antimicrobial susceptibility patterns of *E*. *bruuniana* in the literature. Knowledge on the phylogenetic relationship, genomic traits, and antimicrobial susceptibility patterns provides valuable information on this novel species.

## Supplementary information

Table S1, S2, S3, S4

## Data Availability

The species, strains, and GenBank accession numbers of microorganisms for 16S rRNA gene analysis are listed in Supplementary Table S1. The names of organisms, strains, biosample numbers, bioproject numbers, assembly numbers, isolated origins, and release dates of bacteria used in this study are shown in Supplementary Table S2. All data are available in the NCBI genome sequence repository.
