# Supplementary material for: Genomic Features, Comparative Genomics, and Antimicrobial Susceptibility Patterns of Elizabethkingia bruuniana
Source: Sci Rep. 2019 Feb 19;9:2267. doi: 10.1038/s41598-019-38998-6 (PMC6381114; doi:10.1038/s41598-019-38998-6)
Supplement: Supplementary file 1 — Table S1, S2, S3, S4 [file 41598_2019_38998_MOESM1_ESM.pdf]

Genomic Features, Comparative Genomics, and Antimicrobial Susceptibility Patterns of *Elizabethkingia bruuniana*

Jiun-Nong Lin[1,2,3], Chung-Hsu Lai[1,2], Chih-Hui Yang[4], Yi-Han Huang[1] & Hsi-Hsun Lin[5,6]

[1]School of Medicine, College of Medicine, I-Shou University, Kaohsiung, Taiwan

[2]Division of Infectious Diseases, Department of Internal Medicine, E-Da Hospital, I-Shou University, Kaohsiung, Taiwan

[3]Department of Critical Care Medicine, E-Da Hospital, I-Shou University, Kaohsiung, Taiwan

[4]Department of Biological Science and Technology, Meiho University, Pingtung, Taiwan

[5]General Clinical Research Center, Department of Medical Research, Taipei Veterans General Hospital, Taipei, Taiwan

[6]School of Medicine, National Yang-Ming University, Taipei, Taiwan

*Corresponding author: Jiun-Nong Lin, MD, PhD

Division of Infectious Diseases, Department of Internal Medicine, E-Da Hospital, I-Shou University, Kaohsiung, Taiwan

No. 1, Yida Road, Jiaosu Village, Yanchao District, Kaohsiung, Taiwan

E-mail: jinoli@kmu.edu.tw

Tel: +886-7-6150011-251469

Fax: +886-7-615-0928

Table S1. Species, strains, and accession numbers of 16S rRNA used in this study

**Species**	**Strain**	**GenBank accession number**
Elizabethkingia meningoseptica	ATCC 13253	AJ704540
Elizabethkingia anophelis	R26	EF426425
Elizabethkingia anophelis	EM361-97	NZ_KV757122
Elizabethkingia miricola	W3-B1	AB071953
Elizabethkingia bruuniana	G0146	MH789419
Elizabethkingia bruuniana	EM798-26	CP023746
Elizabethkingia ursingii	G4122	MH789420
Elizabethkingia occulta	G4070	MH789418
Chryseobacterium gleum	NBRC 15054	AB680759
Chryseobacterium indologenes	NBRC 14944	AB517708
Escherichia coli	NBRC 102203	AB681728
Salmonella enterica subsp. *enterica* serovar *Typhimurium*	LT2	AE006468
Pseudomonas aeruginosa	DSM 50071	HE978271
Klebsiella pneumoniae	DSM 30104	AJ233420
Vibrio cholerae	ATCC 14035	EF032498
Proteus hauseri (Proteus vulgaris)	NCTC 4175	DQ885262
Aeromonas caviae	ATCC 15467	X60409
Stenotrophomonas maltophilia	CCUG 41684	GU945534
Acinetobacter calcoaceticus	ATCC 23055	HE651903
Acinetobacter baumannii	ATCC 19606	HE651907

Enterobacter cloacae	DSM 30054	HE978272

Table S2. Information of whole-genome sequences used in this study

**Organism/Name**	**Strain**	**BioSample**	**BioProject**	**Assembly**	**Size (Mb)**	**GC%**	**Genes**	**Proteins**	**Level**
Elizabethkingia miricola (*Elizabethkingia bruuniana*)	BM10	SAMN03398457	PRJNA277897	GCA_000955665.1	4.24252	35.7	3888	3728	Complete Genome
Elizabethkingia miricola (*Elizabethkingia bruuniana*)	EM798-26	SAMN07733282	PRJNA412960	GCA_002557775.1	4.39301	35.7	4029	3877	Complete Genome
Elizabethkingia miricola (*Elizabethkingia bruuniana*)	ATCC 33958	SAMN02989770	PRJNA259681	GCA_000769445.1	4.57811	35.9	4308	4160	Contig
Elizabethkingia miricola	EM_CHUV	SAMN03998138	PRJNA293155	GCA_001483145.1	4.28605	36.0726	3902	3806	Contig
Elizabethkingia miricola	GTC_862 (type strain)	SAMD00016624	PRJNA301708	GCA_001675285.1	4.29381	35.8	4003	3899	Contig
Elizabethkingia miricola	CSID_3000517120	SAMN05254999	PRJNA301708	GCA_002023335.1	4.4291	35.9	4042	3947	Contig
Elizabethkingia miricola	G4121	SAMN05281843	PRJNA301708	GCA_002023475.1	4.42197	35.9	4104	4010	Contig
Elizabethkingia miricola	CSID_3000516464	SAMN05255998	PRJNA301708	GCA_002023515.1	4.18805	35.8	3853	3765	Contig
Elizabethkingia miricola	CSID_3000516998	SAMN05256599	PRJNA301708	GCA_002023575.1	4.36583	36	4073	3967	Contig
Elizabethkingia miricola	G4074 (type strain)	SAMN05277881	PRJNA301708	GCA_002023685.1	4.27041	35.9	3923	3828	Contig
Elizabethkingia miricola	FL160902	SAMN07139315	PRJNA387126	GCA_002738605.1	4.21902	35.7	-	-	Contig
Elizabethkingia miricola	KCTC 12492	SAMEA4051575	PRJEB13258	GCA_900092045.1	4.29997	35.8	4005	3897	Contig
Elizabethkingia miricola	LDVH-337.01	SAMEA4026803	PRJEB14302	GCA_900157035.1	4.15085	35.9	3816	3676	Contig
Elizabethkingia miricola	CIP111047	SAMEA50780668	PRJEB14302	GCA_900157015.1	4.45145	35.9	4197	4098	Scaffold

Elizabethkingia genomosp. 3 (*Elizabethkingia bruuniana*)	G0146	SAMN05277827	PRJNA326741	GCA_002024805.1	4.43352	35.9	4127	4007	Complete Genome
Elizabethkingia genomosp. 3 (*Elizabethkingia bruuniana*)	G0146 (type strain)	SAMN05277827	PRJNA301708	GCA_002023205.1	4.38585	35.8	4086	3981	Contig
Elizabethkingia genomosp. 3 (*Elizabethkingia bruuniana*)	CSID_3000516589	SAMN05256516	PRJNA301708	GCA_002023545.1	4.21084	35.8	3838	3750	Contig
Elizabethkingia genomosp. 3 (*Elizabethkingia bruuniana*)	CSID_3015183685	SAMN05277593	PRJNA301708	GCA_002023765.1	4.3224	35.8	3973	3882	Contig
Elizabethkingia genomosp. 3 (*Elizabethkingia bruuniana*)	G0153	SAMN05277862	PRJNA301708	GCA_002023775.1	4.37908	35.8	4073	3965	Contig
Elizabethkingia genomosp. 3 (*Elizabethkingia bruuniana*)	G4075	SAMN04254558	PRJNA301708	GCA_001521735.1	4.22977	35.8	3914	3817	Scaffold
Elizabethkingia anophelis	R26 (type strain)	SAMN02470677	PRJNA178189	GCA_000331815.1	4.03272	35.40	3746	3643	Complete Genome
Elizabethkingia meningoseptica	KC1913 (type strain)	SAMN04254555	PRJNA301708	GCA_001521755.1	3.85364	36.40	3462	3383	Scaffold
Elizabethkingia genomosp. 4 (*Elizabethkingia ursingii*)	G4122 (type strain)	SAMN04254563	PRJNA301708	GCA_001521765.1	4.3397	35.60	3984	3859	Scaffold
Elizabethkingia genomosp. 4 (*Elizabethkingia occulta*)	G4070 (type strain)	SAMN05277871	PRJNA301708	GCA_002023715.1	4.15401	35.60	3751	3645	Contig

Table S3. Potential virulence factors predicted using the virulence factor database (VFDB)

**Gene id**	**Identity**	**E value**	**Subject id**	**Protein**	**Gene description**
798_26GL000885	41.32	6.00E-94	VFG0036	LPS	bplC - lipopolysaccharide biosynthesis protein
798_26GL000118	45.83	4.00E-68	VFG0077	ClpP	clpP - ATP-dependent Clp protease proteolytic subunit
798_26GL000516	35.49	4.00E-156	VFG0079	ClpC	clpC - endopeptidase Clp ATP-binding chain C
798_26GL001947	32.82	2.00E-84	VFG0116	Alginate	algB - two-component response regulator AlgB
798_26GL003849	31.4	1.00E-18	VFG0119	Alginate	algR - alginate biosynthesis regulatory protein AlgR
798_26GL002789	30.79	1.00E-56	VFG0122	Alginate	algD - GDP-mannose 6-dehydrogenase AlgD
798_26GL001525	33.16	1.00E-65	VFG0130	Alginate	algI - alginate o-acetyltransferase AlgI
798_26GL003331	30.67	1.00E-28	VFG0246	LOS	lgtF - beta-1,4-glucosyltransferase
798_26GL003803	55.14	2.00E-45	VFG0269	Urease	ureA - urease alpha subunit (ureA) (urea amidohydrolase)
798_26GL003806	32.81	2.00E-27	VFG0272	Urease	ureE - urease accessory protein (ureE)
798_26GL003808	64.14	1.00E-104	VFG0274	Urease	ureG - urease accessory protein (ureG)
798_26GL002571	34.4	2.00E-70	VFG0313	LPS	gluP - glucose/galactose transporter (gluP)
798_26GL002401	37.58	5.00E-33	VFG0320	LPS	kdtB - lipopolysaccharide core biosynthesis protein (kdtB)
798_26GL004066	31.69	3.00E-48	VFG0344	HitABC	hitC - iron(III) ABC transporter, ATP-binding protein
798_26GL001922	44.52	6.00E-160	VFG0431	Vi antigen	tviB - Vi polysaccharide biosynthesis protein, UDP-glucose/GDP-mannose dehydrogenase
798_26GL001231	32.12	2.00E-21	VFG0575	MgtBC	mgtC - Mg^{2+} transport protein
798_26GL003027	36.93	9.00E-167	VFG0619	Aerobactin	iutA - receptor-like protein iutA [Shigella flexneri (serotype 2a) 301]
798_26GL001006	33.66	1.00E-76	VFG0670	LPS	gtrB - bactoprenol glucosyl transferase
798_26GL002032	36.15	4.00E-62	VFG0674	SMase	smcL - sphingomyelinase-c

798_26GL000335	32.18	2.00E-52	VFG0679	Capsule	dep/capD - γ-glutamyltranspeptidase
798_26GL003341	30.89	9.00E-25	VFG0869	Dispersin	aatC - AatC ATB binding protein of ABC transporter
798_26GL000890	31.07	6.00E-24	VFG0869	Dispersin	aatC - AatC ATB binding protein of ABC transporter
798_26GL002348	36.26	1.00E-36	VFG0869	Dispersin	aatC - AatC ATB binding protein of ABC transporter
798_26GL002829	30	5.00E-21	VFG0872	Type 1 fimbriae	fimE - Type 1 fimbriae Regulatory protein fimE
798_26GL001929	32.34	9.00E-67	VFG0916	Chu	chuS - Putative heme/hemoglobin transport protein
798_26GL001932	34.3	1.00E-59	VFG0922	Chu	chuU - Putative permease of iron compound ABC transport system
798_26GL000108	30.38	5.00E-21	VFG0934	Enterobactin	entA - 2,3-dihydro-2,3-dihydroxybenzoate dehydrogenase
798_26GL003073	30.68	3.00E-35	VFG0934	Enterobactin	entA - 2,3-dihydro-2,3-dihydroxybenzoate dehydrogenase
798_26GL001943	31.37	2.00E-32	VFG1206	FbpABC	fbpC - iron(III) ABC transporter, ATP-binding protein
798_26GL000094	32.56	2.00E-92	VFG1214	Type IV pili	pilR - two-component response regulator PilR
798_26GL001361	37.93	2.00E-95	VFG1214	Type IV pili	pilR - two-component response regulator PilR
798_26GL002938	44.35	2.00E-67	VFG1248	Flagella	fleQ - transcriptional regulator FleQ
798_26GL002311	47.31	3.00E-113	VFG1300	Capsule	cap8D - capsular polysaccharide synthesis enzyme Cap8D
798_26GL002308	57.18	4.00E-152	VFG1301	Capsule	cap8E - capsular polysaccharide synthesis enzyme Cap8E
798_26GL003580	30.68	2.00E-10	VFG1310	Capsule	cap8N - capsular polysaccharide synthesis enzyme Cap8N
798_26GL002302	31.86	5.00E-35	VFG1343	Capsule	cpsN - glycosyl transferase CpsN(V)
798_26GL003658	36.32	1.00E-34	VFG1344	Capsule	cpsM - polysaccharide biosynthesis protein CpsM(V)
798_26GL000190	31.98	8.00E-38	VFG1390	MprAB	mprA - hypothetical protein Rv0981
798_26GL003363	32.44	2.00E-53	VFG1390	MprAB	mprA - hypothetical protein Rv0981
798_26GL001772	33.18	8.00E-52	VFG1390	MprAB	mprA - hypothetical protein Rv0981

798_26GL001370	34.22	3.00E-42	VFG1390	MprAB	mprA - hypothetical protein Rv0981
798_26GL001431	35.34	6.00E-60	VFG1390	MprAB	mprA - hypothetical protein Rv0981
798_26GL000086	31.4	2.00E-21	VFG1395	MgtC	mgtC - mgtC
798_26GL001791	36.16	6.00E-48	VFG1395	MgtC	mgtC - mgtC
798_26GL001174	33.74	5.00E-29	VFG1404	AhpC	ahpC - ahpC
798_26GL001165	35.5	6.00E-67	VFG1405	SigA	sigA - sigA
798_26GL003523	50.47	9.00E-38	VFG1416	PanC/PanD	panD - panD
798_26GL002277	40.21	4.00E-47	VFG1417	PanC/PanD	panC - panC
798_26GL001915	30	2.00E-42	VFG1444	AslA	aslA - putative arylsulfatase
798_26GL001526	41.26	2.00E-36	VFG1864	Mip	mip - macrophage infectivity potentiator (Mip)
798_26GL000580	39.18	7.00E-49	VFG1867	SodB	sodB - superoxide dismutase
798_26GL001218	39.59	2.00E-55	VFG1867	SodB	sodB - superoxide dismutase
798_26GL002823	43.88	1.00E-61	VFG1867	SodB	sodB - superoxide dismutase
798_26GL001508	39.48	2.00E-83	VFG1971	Capsule	kpsF - KpsF protein
798_26GL001317	32.14	4.00E-18	VFG2008	Flagella	motB - flagellar motor protein MotB
798_26GL003326	30.6	7.00E-28	VFG2224	LPS	wbkA - mannosyltransferase
798_26GL000886	40.84	1.00E-103	VFG2361	O-antigen	galE - UDP-glucose 4-epimerase
798_26GL002328	45.62	7.00E-115	VFG2364	O-antigen	fcl - GDP-flucose synthetase
798_26GL002327	58.61	5.00E-169	VFG2365	O-antigen	gmd - GDP-mannose 4,6-dehydratase

Table S4. Potential antibiotic resistance genes identified using the Antibiotic Resistance Genes Database (ARDB) BLAST Server

**Gene id**	**Identity**	**E value**	**Subject id**	**Antibiotic Resistance**	**Description**
798_26GL001208	30	6.00E-39	ardb_2322	Bacitracin	ABC transporter system, bacitracin efflux pump.
798_26GL001461	30	3.00E-24	ardb_2858	Bacitracin	--
798_26GL001650	30.13	6.00E-37	ardb_115	Vancomycin	VanE type vancomycin resistance operon genes, which can synthesize peptidoglycan with modified C-terminal D-Ala-D-Ala to D-alanine--D-serine.
798_26GL003067	30.46	3.00E-53	ardb_2749	Vancomycin	VanG type vancomycin resistance operon genes, which can synthesize peptidoglycan with modified C-terminal D-Ala-D-Ala to D-alanine--D-serine.
798_26GL002835	30.51	2.00E-33	ardb_1732	Vancomycin, teicoplanin	VanA type vancomycin resistance operon genes, which can synthesize peptidoglycan with modified C-terminal D-Ala-D-Ala to D-alanine--D-lactate.
798_26GL000126	30.95	3.00E-34	ardb_2452	Sulfonamide	Sulfonamide-resistant dihydropteroate synthase, which cannot be inhibited by sulfonamide.
798_26GL000603	31	9.00E-36	ardb_2470	Vancomycin, teicoplanin	VanA type vancomycin resistance operon genes, which can synthesize peptidoglycan with modified C-terminal D-Ala-D-Ala to D-alanine--D-lactate.
798_26GL004049	31.09	9.00E-62	ardb_2640	Macrolide	Resistance-nodulation-cell division transporter system. Multidrug resistance efflux pump. Macrolide-specific efflux system.
798_26GL000821	31.3	2.00E-62	ardb_1951	Chloramphenicol	Major facilitator superfamily transporter,

					chloramphenicol efflux pump.
798_26GL002854	31.36	1.00E-06	ardb_2497	Vancomycin, teicoplanin	VanA type vancomycin resistance operon genes, which can synthesize peptidoglycan with modified C-terminal D-Ala-D-Ala to D-alanine--D-lactate.
798_26GL002400	31.76	3.00E-51	ardb_2028	Vancomycin	VanG type vancomycin resistance operon genes, which can synthesize peptidoglycan with modified C-terminal D-Ala-D-Ala to D-alanine--D-serine.
798_26GL003582	32	2.00E-27	ardb_654	Vancomycin	VanG type vancomycin resistance operon genes, which can synthesize peptidoglycan with modified C-terminal D-Ala-D-Ala to D-alanine--D-serine.
798_26GL000111	32.01	2.00E-53	ardb_1853	Bacitracin	ABC transporter system, bacitracin efflux pump.
798_26GL001431	32.63	2.00E-41	ardb_115	Vancomycin	VanE type vancomycin resistance operon genes, which can synthesize peptidoglycan with modified C-terminal D-Ala-D-Ala to D-alanine--D-serine.
798_26GL001118	32.82	7.00E-40	ardb_1739	Kasugamycin	Specifically dimethylates two adjacent adenosines in the loop of a conserved hairpin near the 3'-end of 16S rRNA in the 30S particle. Its inactivation leads to kasugamycin resistance.
798_26GL003751	32.98	5.00E-21	ardb_2747	Cephalosporin	Class C beta-lactamase. This enzyme breaks the beta-lactam antibiotic ring open and deactivates the molecule's antibacterial properties.
798_26GL000656	34.05	2.00E-56	ardb_2990	Bacitracin	ABC transporter system, bacitracin efflux pump.

798_26GL002404	34.15	3.00E-40	ardb_13	Trimethoprim	Group A drug-insensitive dihydrofolate reductase, which cannot be inhibited by trimethoprim.
798_26GL000620	34.26	1.00E-16	ardb_1205	Multiple antimicrobials	Small Multidrug Resistance (SMR) protein family. Multidrug resistance efflux pump, which consists of two proteins.
798_26GL001823	34.98	1.00E-49	ardb_998	Penicillin	Class A beta-lactamase. This enzyme breaks the beta-lactam antibiotic ring open and deactivates the molecule's antibacterial properties.
798_26GL001370	35.14	1.00E-39	ardb_1550	Vancomycin	VanB type vancomycin resistance operon genes, which can synthesize peptidoglycan with modified C-terminal D-Ala-D-Ala to D-alanine-D-lactate.
798_26GL001069	35.47	3.00E-82	ardb_1016	Tetracycline	Major facilitator superfamily transporter, tetracycline efflux pump.
798_26GL001517	36.11	1.00E-15	ardb_1267	Multiple antimicrobials	Small Multidrug Resistance (SMR) protein family. Multidrug resistance efflux pump, which consists of two proteins.
798_26GL000702	36.21	2.00E-72	ardb_916	Cephalosporin, penicillin	Class A beta-lactamase. This enzyme breaks the beta-lactam antibiotic ring open and deactivates the molecule's antibacterial properties.
798_26GL000324	38.98	1.00E-69	ardb_916	Cephalosporin, penicillin	Class A beta-lactamase. This enzyme breaks the beta-lactam antibiotic ring open and deactivates the molecule's antibacterial properties.

798_26GL001121	39.43	2.00E-67	ardb_2724	Bacitracin	Undecaprenyl pyrophosphate phosphatase, which consists in the sequestration of undecaprenyl pyrophosphate.
798_26GL000331	40	5.00E-29	ardb_1787	Aminoglycoside	Multidrug resistance efflux pump.
798_26GL000052	55.35	8.00E-160	ardb_2144	Tetracycline	NADP-requiring oxidoreductase, an enzyme that can modify tetracycline. Mechanism detail unknown.
798_26GL002831	63.41	3.00E-108	ardb_2771	Chloramphenicol	Group B chloramphenicol acetyltransferase, which can inactivate chloramphenicol. Also referred to as xenobiotic acetyltransferase.
